# The Effect of Polyphenols on Protein Degradation Pathways: Implications for Neuroprotection

**DOI:** 10.3390/molecules22010159

**Published:** 2017-01-19

**Authors:** Parvana Hajieva

**Affiliations:** Department of Pathobiochemistry, University Medical Center of the Johannes Gutenberg University, Duesbergweg 6, 55099 Mainz, Germany; hajieva@uni-mainz.de; Tel.: +49-6131-392-4552; Fax: +49-6131-392-4743

**Keywords:** protein oxidation, proteostasis, protein degradation, proteasome, macroautophagy, chaperone-mediated autophagy, neuroprotection

## Abstract

Human neurodegenerative diseases are accompanied by accumulation of heavily oxidized and aggregated proteins. However, the exact molecular reason is not fully elucidated yet. Insufficient cellular protein quality control is thought to play an important role in accumulating covalently oxidized misfolded proteins. Pharmacologically active polyphenols and their derivatives exhibit potential for preventive and therapeutic purposes against protein aggregation during neurodegeneration. Although these compounds act on various biochemical pathways, their role in stabilizing the protein degradation machinery at different stages may be an attractive therapeutical strategy to halt the accumulation of misfolded proteins. This review evaluates and discusses the existing scientific literature on the effect of polyphenols on three major protein degradation pathways: chaperone-mediated autophagy, the proteasome and macroautophagy. The results of these studies demonstrate that phenolic compounds are able to influence the major protein degradation pathways at different levels.

## 1. Introduction

Neuronal proteins undergo continuous turnover at distinct rates, and this degradative process plays an important role in protein homeostasis, also called proteostasis. The components of the proteostasis machinery/network decide about the fate of damaged proteins, which either ensure their refolding into original stable conformation or eliminate them from the cell through proteolysis [1]. These strict protein quality control mechanisms coordinate the rate of protein synthesis through degradation of damaged proteins, which, in turn, is crucial to maintaining basic cellular processes such as regulation of cell cycle, gene transcription, and metabolic pathways, since the major regulators of these processes are proteins and rate limiting enzymes with a limited half-life.

There are three major coordinated cellular protein degradation pathways: (i) the chaperone-mediated pathway, also called chaperone-mediated autophagy; (ii) the ubiquitin–proteasome degradation pathway; and (iii) the lysosome–autophagy system, also called macroautophagy [2]. Under normal circumstances, proteins with abnormal conformations are rapidly degraded [3,4]. However, insufficient refolding or degradation of damaged, aggregation-prone proteins can lead to their cellular accumulation.

Neurons are post-mitotic, bipolar cells, and, thus, thought to be particularly vulnerable to toxic protein aggregates. Indeed, neuronal accumulation of heavily oxidized, aggregation-prone proteins plays a causal role in the pathogenesis of age-associated neurodegenerative diseases, such as Alzheimer’s disease [5,6] and Parkinson’s disease [7]. In fact, they are generically considered as proteinopathies or protein conformational diseases [8]. The term of proteinopathy refers to pathologies associated with an intra- or extracellular accumulation of misfolded or aggregated proteins. This is thought to be one of the major hallmarks of neurodegenerative disorders. Despite of the involvement of distinct proteins in their pathology, the neurological disorders such as Alzheimer’s disease, Parkinson’s disease, amyotrophic lateral sclerosis, frontotemporal dementia, prion disease, age-related macular degeneration, and Huntington’s disease are the most prominent proteinopathies of the central nervous system [9]. Although the exact trigger for these proteinopathies is not known yet, the altered or insufficient proteostasis is thought to play an important role here. This is most probably due to a compromised function of neuronal proteostasis under basal and chronic stress conditions [10]. In fact, a gradual decline in cellular proteostasis capacity results in the accumulation of misfolded (or oxidized) proteins, thus leading to aggregate deposition with toxic effects and cell death [11,12]. The importance of coordinated function of proteasome, chaperone-mediated autophagy as well as macroautophagy in the pathogenesis of proteinopathies was demonstrated in various preclinical animal models. In fact, the disturbance of the coordinated action of this network leads to accumulation of insoluble protein aggregates, which ultimately leads to neurodegeneration [13]. For instance, neuron specific knockdown of autophagic key genes ATG5 and ATG7 was sufficient for inducing the accumulation of protein aggregates, axonal degeneration and neuronal cell death [14]. Autophagy is a key regulator of intracellular aggregation-prone proteins, as it is demonstrated in most prominent proteinopathies such as polyglutamine-expanded huntingtin in Huntington’s disease [15]; mutant TDP43 in amyotrophic lateral sclerosis [16]; mutant forms of α-synuclein in Parkinson’s disease [17]; and mutant tau proteins in various types of dementia including Alzheimer’s disease [18]. All of these studies highlight the importance of an efficient neuronal protein degradation network.

Pharmacological targeting of the proteostasis network to maintain the coordinated intracellular protein homeostasis may be an important strategy to hamper (the progression of) age-related neurodegeneration. In fact, independently of the trigger mechanism of proteinopathies, the increase in degradation of these misfolded, aggregation-prone proteins may slow down the progression of diseases considered as proteinopathies. 

Strong evidence suggests that natural dietary compounds exhibit health promoting effects (for review, see [19]). Flavonoid and non-flavonoid polyphenols, due to their abundance in the plant kingdom, represent the largest group of phytochemicals. In fact, there are more than 8000 existing natural polyphenols whose chemistry is very diverse; however, their major common structural property is the presence of more than one phenol group per molecule. Strong evidence suggests that foods or beverages rich in polyphenols increase blood serum antioxidant levels and thereby contribute to preventing oxidative stress-induced cell damage [20,21]. Flavonoids, in contrast to non-flavonoids, represent the largest group of polyphenols. They are natural substances present in fruit and vegetables as well as processed foods, including olive oil, red wine, and tea; the total daily intake of polyphenols mainly from fruit and vegetables is approximately 1 g per day, depending on dietary preferences [22].

Widely investigated flavonoid polyphenols are quercetin **10**, rutin **14** and catechins **1**–**8**. Numerous publications report the neuroprotective effects of catechins, major constituents of green tea and cacao beans, in different models of neurodegeneration. Rutin **14**, a glucoside of quercetin, is a therapeutically attractive flavonoid due to its relatively high blood–brain barrier permeability. Numerous studies have revealed beneficial effects of rutin **14** in different in vitro and in vivo models of neurodegeneration. However, most of these effects were assigned to their ability to restore the neuronal oxidant/antioxidant balance as well as to reduce the aggregation potential of proteins [22].

Well-known non-flavonoid polyphenols are resveratrol **20**, ellagic acid **21** and its derivatives, and lignans such as arctigenin **17**, curcumin **18** and rosmarinic acid **19**. All of these compounds are of plant origin: the highest level of resveratrol can be found in grapes and red wine; ellagic acid **21** and its derivatives are abundant in different berries, pomegranates and walnuts, and the bound forms of lignans are found in flaxseeds, sesame seeds, and different grains. Curcumin **18** is a non-flavonoidic polyphenol commonly known as turmeric; the powdered root of *Curcuma longa* possesses strong antioxidant properties. Rosmarinic acid **19** is a dimer of caffeic acid found in a variety of plants including many culinary herbs, such as basil, rosemary, thyme and peppermint, while ellagic acid **21** is a dimer of gallic acid found in gallnuts [23].

It is known from literature that many investigations with prominent members of polyphenols have demonstrated a reverse influence of these substances on different proteinopathies. Various epidemiological investigations reported a strong inverse correlation between a diet rich in polyphenols and/or antioxidants and the incidence of neurodegenerative disease accompanied by proteinopathies—in particular, Alzheimer’s disease [24,25]. An investigation on human subjects above 65 years of age demonstrated a strong inverse relationship between flavonoid rich diet (fruit, vegetables, red wine, and green tea) and the risk of dementia [26]. Numerous studies, for example, report that the daily consumption of the *Ginkgo biloba* extract EGb 761 leads to an improved cognitive performance in patients with Alzheimer’s disease [27,28,29]. To date, the major pharmacologically active constituents of the widely used *Ginkgo biloba* extract EGb 761 are flavonol glycosides (24%), the main constituents of which are kaempferol **9**, quercetin **10** and isorhamnetin **11**. The remaining fraction of this extract comprises terpene trilactones (6%) as well as a variety of unknown substances (about 13%) [30,31]. Furthermore, a cross national epidemiological study comparing the incidence of Alzheimer’s disease in Indian and American communities reported a significantly lower prevalence of this disease in the Indian community. This effect was assigned to a high dietary consumption of curcumin **18** [32].

A large number of epidemiological investigations also reported a strong inverse correlation between the dietary intake of foods rich in polyphenols and/or antioxidants and the incidence of Parkinson’s diseases [33,34]. High tea consumption was reported to be associated with a lower risk of Parkinson’s disease [35,36].

Taken together, all of these epidemiological and preclinical results demonstrate beneficial effects of flavonoids and polyphenols in neurodegenerative diseases. These effects are suggested to be primarily exerted through their antioxidant properties and their influence on stress response through Nrf-2 signaling, which are directed towards the induction of antioxidant potential as well as an enhancement of cellular stress-response, which, in turn, can prevent cellular damage by increasing its antioxidant defense. New evidence suggests that, apart from being antioxidative, polyphenols can also directly or indirectly strengthen the degradation of misfolded and damaged proteins. This can be achieved by increasing the activity and efficiency of the cellular protein degradation machinery [13].

Since, in fact, the common pathological mechanism shared by prominent neurodegenerative diseases is the accumulation of insoluble protein aggregates, boosting the degradation of such aggregates by polyphenols is becoming an attractive prevention and therapeutic strategy. The coordinated action of all three degradation pathways enhanced or supported by polyphenols may help neurons to keep the balance between the formation of misfolded proteins and their degradation.

**Figure 1:**
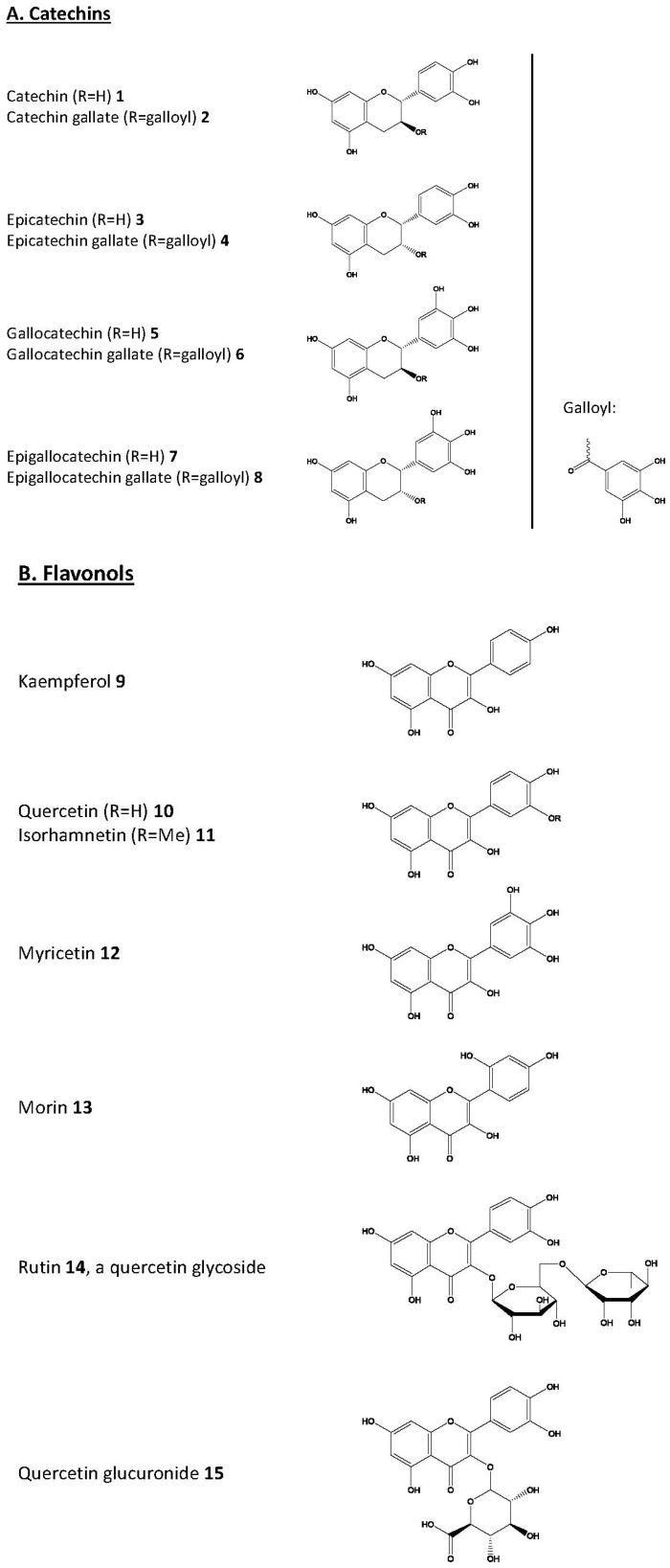

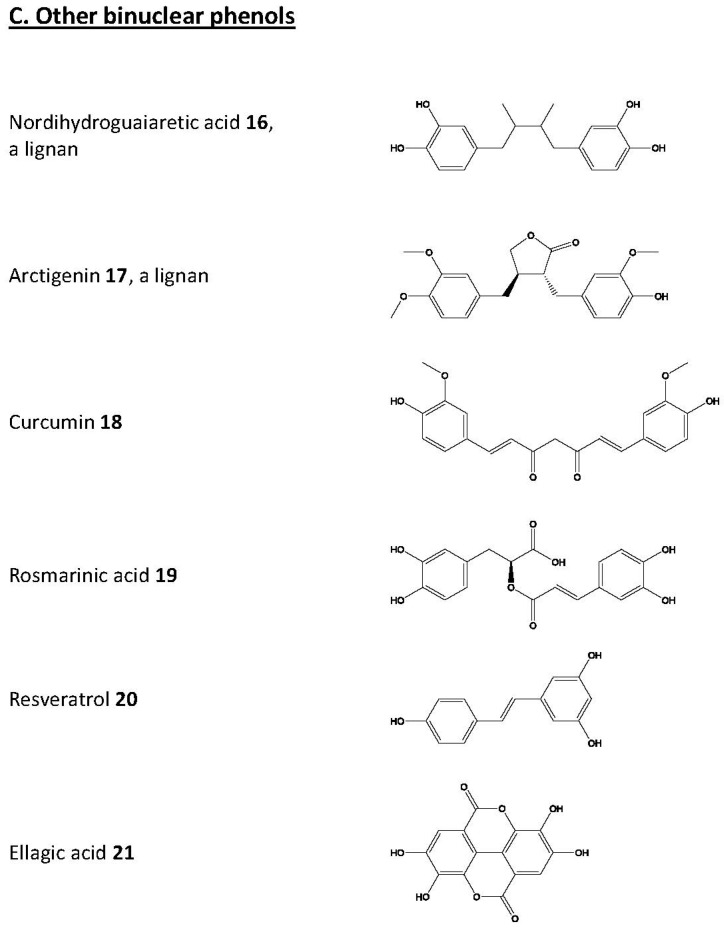

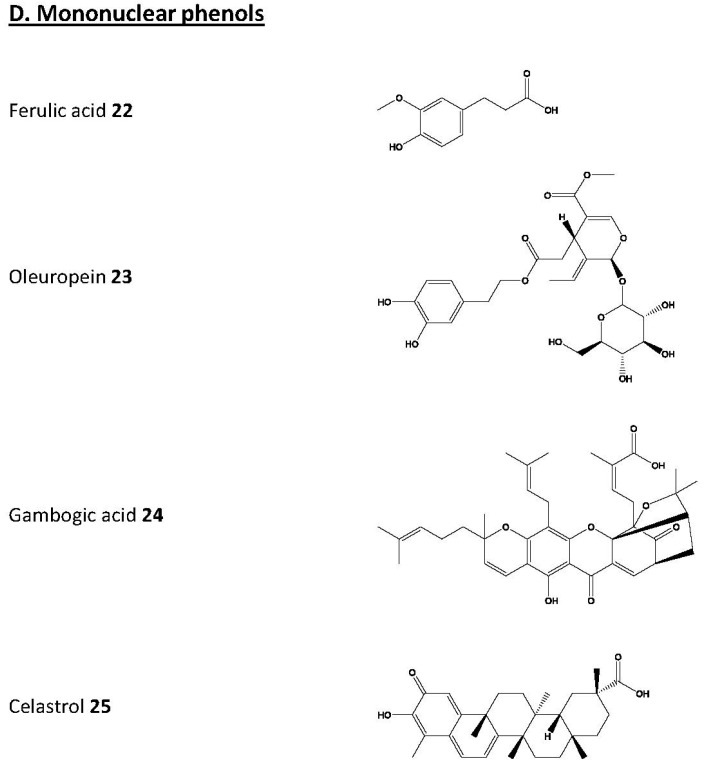
Chemical structures of the compounds discussed in the text. (**A**) catechins; (**B**) flavonols; (**C**) binuclear phenolic structures and (**D**) mononuclear phenolic compounds.

This review summarizes and discusses key studies demonstrating the beneficial effects of polyphenols (Figure 1) on the main cellular protein degradation pathways: chaperone-mediated autophagy, the proteasome, and macroautophagy.

## 2. Effect of Polyphenols on Chaperone-Mediated Autophagy

Under normal physiological conditions, proteins undergo different conformational changes during their lifetime. Each step is assisted by chaperones and includes de novo folding, assembly and disassembly, transport across membranes, and targeting for degradation [37]. The expression of many chaperones is strongly induced under conditions of oxidative stress and heat shock challenges. Therefore, they are considered to be stress proteins or heat shock proteins (HSPs). Various group members of the chaperone network were named according to their molecular weight: Hsp40s, Hsp60s, Hsp70s, Hsp90s, Hsp100s, and the small Hsps. The molecular chaperone network is classified into different groups on the basis of sequence homology [38,39].

Preventing age-related neurodegenerative diseases by boosting the chaperone network might be a promising strategy and could be achieved through targeting it at its three levels—biogenesis, conformational maintenance, and degradation—either individually or in combination. Various small molecular mass compounds called chemical chaperones have been proven to increase protein stability in vitro [40]. However, those unnatural compounds exhibit a high toxic potential, rendering them unsuitable for in vivo application. Consequently, using dietary compounds which can restore the chaperone network appears to be an attractive strategy. It is well established that the beneficial effects of polyphenols can be exerted through their antioxidant properties. The phenolic group can accept an electron and thereby act as a chain-breaking antioxidant [41]. Moreover, polyphenols can directly induce cellular stress response by increasing chaperone levels. In fact, resveratrol **20** as a prominent polyphenol was shown to induce the acute heat shock response by upregulating chaperones like Hsp70 [42].

Curcumin **18**, abundant in yellow curry spices widely used by Indian as well as other South and East Asian populations, shows cytoprotective effects and induces nuclear translocation of HSF1 [43], while furthermore displaying anti-inflammatory and antioxidant activities.

Additionally, the stochastic conformational analysis of green tea catechins, (−)-epicatechin gallate **4**, (−)-epigallocatechin **7**, and (−)-epigallocatechin gallate **8**, revealed functional and structural similarities between catechins and chaperones [44], marking them as promising candidates to boost cellular protein stability.

Various mononuclear phenols such as gambogic acid **24** were also identified as direct HSP activators [45]. Isolated human neuroglia cells treated with adaptogens extracted from roots of *Eleutherococcus senticosus*, *Schisandra chinensis* berry and *Rhodiola rosea* also demonstrated an enhancement of HSP70 mediated by an increase in its expression [46].

All these research data suggest that polyphenols could increase the cellular chaperone levels and prevent neurodegeneration. 

## 3. Effect of Polyphenols on the Ubiquitin–Proteasome Degradation Pathway

The proteasome is the major enzymatic proteolytic machinery in charge of maintaining efficient protein turnover or proteostasis. It is a cylindrically shaped muticatalytic enzyme complex called 26S, which, in turn, consists of two major 20S- and 19S- subcomplexes. The subcomplex 20S core particle contains the protease subunits, while the 19S regulatory particle regulates the function of the former.

The 20S core particle is a cylinder-shaped structure that consists of four rings containing seven α- and seven β-subunits each. Two inner β-rings contain the proteolytic active sites that are directed towards the proteolytic chamber. One or two 19S regulatory particles are attached to the surface of the outer 20S core particle α-rings to form the 26S proteasome holoenzyme. Proteins targeted for degradation are allowed to access the outer 20S core particle α-rings through a narrow aperture to the catalytic/proteolytic interior of the proteasome. It is believed that α-subunits influence the activity and specificity of the 20S core particle by interacting or binding different regulators. Although β1, β2 and β5 have a common mode of action, they possess three different substrate specificities: (i) chymotrypsin-like activity, hydrolyzing proteins after acidic or basic amino acids; (ii) trypsin-like activity, cleaving hydrophobic amino acids; (iii) caspase-like or peptidyl-glutamyl peptide-hydrolyzing (PHGH)-like activity, hydrolyzing proteins by cleaving after acidic peptide bonds [47].

The 19S regulatory particle itself consists of multisubunit substructures: a lid and a base. These constituents are responsible for protein substrate recognition, deubiquitination, and the switch of the protein substrate to the 20S core particle [48,49]. Damaged proteins with an abnormal structure, e.g., unfolded, misfolded, and/or highly oxidized proteins, are primarily degraded by the proteasome. Accumulating evidence suggests a significant decline in proteasomal function with aging and in age-related diseases [50,51]. Therefore, restoring/maintaining proteasomal function remains a promising therapeutical target to delay the onset of age-related neurodegeneration.

Two approaches could be pursued to achieve this goal: (i) the enhancement of proteasome capacity by genetic manipulation and (ii) a pharmacologically induced increase of proteasomal activity by employing natural and synthetic compounds.

Natural polyphenols and their derivatives have been reported to enhance proteasome activity in primary fibroblasts. Phenolic and flavonoid components of bee pollen are reported to enhance the chymotrypsin-like activity of the proteasome in HFL-1 human embryonic fibroblasts [52]. Furthermore, human IMR90 and WI-38 embryonic fibroblasts continuously cultivated with oleuropein **23**—a natural compound isolated from *Olea europea* leaves, olives, and olive oil—showed increased proteasome activity, reduced protein carbonylation, and extended life span [33].

Quercetin **10**, widely distributed in nature, is one of the most frequently investigated compounds. It has been shown to be neuroprotective in various in vitro and in vivo systems as well as in human studies. One of the mechanisms of quercetin **10** action is thought to be exerted through the increase of nuclear factor (erytroid-derived 2)-like 2 (Nrf 2) protein levels. Keap1-Nrf-2 is a transcription factor, being a major cellular stress response pathway, and enhances the expression of many antioxidant and phase II drug-metabolizing enzymes [53]. Moreover, quercetin **10** was shown to induce the expression of chaperones and proteasome subunits [54].

## 4. Effect of Polyphenols on Macroautophagy

Macroautophagy is an evolutionary conserved intracellular process that is used by the cell to degrade dysfunctional, aggregated proteins as well as damaged cell organelles. Under physiological conditions, autophagy occurs on a basal level; however, it is upregulated upon cellular stress conditions such as nutrient starvation, and it can also be activated upon oxidative stress. Strong evidence suggests an accumulation of oxidized, cross-linked and aggregated proteins in age-related neurodegeneration [6,55]. Such heavily damaged and aggregated proteins are thought to be mainly degraded by macroautophagy due to their proteasome-inhibiting effects [56]. Hence, an altered and disturbed macroautophagy is also believed to play an important role in proteinopathies. Accumulation of damaged mitochondria as well as insufficient autophagic degradation of protein aggregates causes cell damage in age-related neurodegenerative diseases, such as Alzheimer’s and Parkinson’s diseases. Moreover, numerous studies indicate that the neuronal cell damage occurring in these two most prominent neurodegenerative diseases is accompanied by an impaired autophagic degradation of amyloidogenic Aβ or α-synuclein, respectively [57,58]. Consequently, targeting macroautophagy to prevent neurodegeneration has become an attractive strategy. [59]. In addition to their influence on chaperone-mediated autophagy and proteasome activity, the beneficial effects of natural bioactive polyphenols such as resveratrol **20**, epigallocatechin-3-gallate (EGCG) **8**, curcumin **18**, morin **13**, quercetin **10**, and oleuropein aglycone **23**, on the prevention of cell damage and cell death, are thought to be mediated by modulating macroautophagy. There is considerable evidence showing that polyphenolic compounds ameliorate cognitive defects in models with Alzheimer’s disease. Five phenolic compounds—myricetin **12**, nordihydroguaiaretic acid **16**, curcumin **18**, ferulic acid **22** and rosmarinic acid **19**—demonstrated a significant decreasing effect on Aβ deposition in the brain of Alzheimer’s disease transgenic mice models in five different independent studies [60,61,62]. The observed positive effect of polyphenols investigated in these studies was associated with reduced processing of amyloidβ, which is the main component of extracellular plaques. A polyphenol-supplemented diet was shown to reduce neuro-inflammation in mice as well as reduce reactive oxygen species production in a Drosophila Parkinson’s disease model [63,64]. Interestingly, the beneficial effects of the investigated polyphenols were, for example, a decline in protein aggregation and neuroinflammation, or a decrease in levels of damaged mitochondria. Parallel studies demonstrate an inverse relationship between decline in autophagic activity and increased amyloidβ deposition in mice models with Alzheimer’s disease [65]. Hence, it is plausible to assume that the induction of autophagy by polyphenols could be partially responsible for their beneficial neuroprotective effects. Recent studies in TgCRND8 (Tg) mice, a double transgenic mouse model with Alzheimer’s disease, demonstrate that a mix of polyphenols ameliorates behavioral performance and neuropathology by significantly reducing Aβ42 and pE-3Aβ plaque areas as well as numbers, while, in parallel, significantly inducing autophagy [66]. Feeding these mice with the polyphenol oleuropein aglycone **23** propagated a high autophagic response, as was shown by an increased expression of autophagic markers as well as increased autophagic and lysosomal activity [67].

Furthermore, a polyphenol-enriched fraction from leaves of *Corema album* was shown to reduce α-Synuclein toxicity and aggregation by promoting autophagic activity and reducing oxidative stress in cellular models of Parkinson’s disease [68]. Quercetin **10**, in turn, dose-dependently reduced Amyloid-beta (Aβ₁₋₄₂)-induced paralysis in *Caenorhabditis elegans* by decreasing the amount of aggregated proteins [69]. Similarly, quercetin **10** antagonized the high glucose-induced damage of Schwann cells by inducing autophagy [70]. Resveratrol **20** was demonstrated to induce autophagy by inducing Adenin mononucleotide phosphate kinase (AMPK) signaling in a double transgenic Amyloid precursor protein (APP)/Presenillin1 (PS1) mouse model with Alzheimer’s disease [71]. Another independent study reported neuroprotective effect by a binuclear phenol arctigenin **17** in the same animal model [72].

## 5. Conclusions

Polyphenols found in the plant kingdom can target the structure, assembly, and functioning of the cellular protein quality control machinery called proteostasis. This can be achieved at three different levels: (i) at the genomic level, by increasing the transcription of genes that code for proteins, which are constituents of the protein quality control machinery; (ii) at the expression level, by regulating efficient ribosomal translation of these proteins, their post translational modifications, as well as their proper folding and assembly into multienzymatic complexes; (iii) at the functional level, by increasing the activity of all enzymatic reactions regulating protein quality control. Consequently, targeting the protein degradation machinery through employment of natural polyphenols remains a promising therapeutic approach to delaying the onset of age-related neurodegeneration. Although more and more preclinical investigations tackling the effect of polyphenols on protein degradation pathways are emerging, results of future epidemiological and clinical studies, in particular, will open new avenues on developing novel therapeutical strategies to combat age-related proteinopathies. Thus far, numerous preclinical investigations have reported considerable beneficial effects of a number of polyphenols against proteinopathies. However, the major limitations of using these compounds in humans—and thus, of their clinical applicability—are their low bioavailability limited resorption due to weak solubility in water as well as their poor blood–brain barrier permeability [73,74]. Recent research with red wine identified specific polyphenol metabolites in the brain, such as quercetin glucuronide **15**, providing evidence that the observed beneficial effects of polyphenols are exerted by certain brain-targeting polyphenol metabolites [75]. Another study using different specific grape-derived polyphenolic preparations reports that certain compounds from this mixture, such as catechin **1** and epicatechin **3**, are detectable in the brain only in their monomeric forms [76]. Future studies should focus on using monomeric forms of single bioactive polyphenols or herbal formulae with the highest concentration of these bioactive and blood–brain barrier permeable compounds. Apart from using bioactive polyphenols, approaches for enhancing polyphenol bioavailability, such as their encapsulation as phospholipid nanoparticles, incorporation with biodegradable polymers, or their modifications to improve pharmacokinetics by using adjuvants as absorption enhancers, should be pursued [77]. Hence, future studies in this direction may help to overcome the limitations of using polyphenols in clinical trials. Moreover, the identification of the exact molecular impact of certain blood–brain barrier permeable polyphenols on the neuronal protein degradation machinery may provide a mechanistic clue to understanding their so far reported beneficial and neuroprotective effects.

The question about the toxicity or side effects of high dosage of polyphenols is also frequently addressed. Three possible mechanisms are proposed, which may explain the toxicity of polyphenols: (i) based on pro-oxidant effects due to their radical scavenging properties; (ii) induction of the acute stress response; and (iii) excessive biotransformation in the liver. The pro-oxidant activities are linked to their catechol structure, the chemical characteristic of which is the generation of superoxide anion, which, in turn, might lead to the generation of quinone counterparts (for review, see [78]). Moreover, excessive biotransfomation of polyphenols as xenobiotics by the liver may cause hepatotoxicity. The same may be true for their nephrotoxicity. Therefore, an optimal dosage of single compounds or polyphenol-enriched extracts may increase their therapeutic success.

It is suggested that the neuroprotective ability of polyphenols is mainly attributed to the presence of one or more phenol groups [79,80]. Thus, further approaches based on chemical drug design, concentrating on improving different endogenous and synthetic compounds with similar structures, can be pursued for their favorable effects [81,82]. Accordingly, research should also focus on developing novel synthetic polyphenol compounds based on structure optimization favoring blood–brain barrier permeability in order to overcome these limitations.
